# Analysis of the genetic basis of height in large Jewish nuclear families

**DOI:** 10.1371/journal.pgen.1008082

**Published:** 2019-07-08

**Authors:** Danny Zeevi, Joshua S. Bloom, Meru J. Sadhu, Adi Ben Yehuda, David Zangen, Ephrat Levy-Lahad, Leonid Kruglyak

**Affiliations:** 1 Department of Human Genetics, University of California, Los Angeles, Los Angeles, California, United States of America; 2 Department of Biological Chemistry, University of California, Los Angeles, Los Angeles, California, United States of America; 3 Howard Hughes Medical Institute, University of California, Los Angeles, Los Angeles, California, United States of America; 4 Medical Genetics Institute, Shaare Zedek Medical Center, Jerusalem, Israel; 5 Faculty of Medicine, Hebrew University of Jerusalem, Jerusalem, Israel; 6 Division of Pediatric Endocrinology, Hadassah Hebrew University Medical Center, Jerusalem, Israel; University of Chicago, UNITED STATES

## Abstract

Despite intensive study, most of the specific genetic factors that contribute to variation in human height remain undiscovered. We conducted a family-based linkage study of height in a unique cohort of very large nuclear families from a founder (Jewish) population. This design allowed for increased power to detect linkage, compared to previous family-based studies. Loci we identified in discovery families could explain an estimated lower bound of 6% of the variance in height in validation families. We showed that these loci are not tagging known common variants associated with height. Rather, we suggest that the observed signals arise from variants with large effects that are rare globally but elevated in frequency in the Jewish population.

## Author summary

Rare variants with large effects have been suggested to account for some of the missing heritability of height, but detection of such variants in genome-wide association studies (GWAS) requires very large sample sizes due to their low frequencies. Here, we designed a unique study of height, in which we sought to increase the effective frequency of rare variants in our sample. This was done by assembling a cohort of very large nuclear families, with an average of 12 and a maximum of 20 siblings per nuclear family. In this design, any variant segregating in our cohort has a minimum expected frequency of ~1%, regardless of its frequency in the general population. In addition, we recruited all participants from a founder (Jewish) population, in which some variants that are rare in a cosmopolitan population can rise to high frequencies. To further increase power, we developed methods to obtain highly accurate height measurements, genotype calls, and inheritance pattern reconstructions. These factors allowed us to detect many more QTLs than previous family-based studies of height, despite a modest sample size of only 397 participants. The approach described in this paper provides insights into the genetic basis of height and the roles of population-specific vs. cosmopolitan variants, and may serve as a complement to GWAS for genetic investigations of other complex traits.

## Introduction

Height is a classic genetically complex quantitative trait with high heritability (~80%-90% [1,2]). Despite intensive study, the genetic basis of variation in height remains mostly unexplained. Genome-wide association studies (GWAS) in hundreds of thousands of individuals have identified hundreds of common variants significantly associated with height [3,4]. However, the individual effect sizes of these variants are small, and all variants identified to date jointly explain only ~20% of the heritability of height. One proposed explanation for the gap between the overall heritability of height and that explained by common variants is the contribution of rare genetic variants with large phenotypic effects [5]. Recent studies lend support to this idea by identifying such variants and showing that their effect sizes are inversely related to their frequencies [6,7]. Several examples of large-effect variants that are rare globally but are more common in certain founder populations have been reported for height (in Sardinians [8] and in Puerto Ricans [9]) and diabetes (in Greenlanders [10]), however, identifying associations between rare variants and traits of interest typically requires very large sample sizes [11]. For instance, 750,000 participants were required to identify 32 rare variants (those with frequency <1%) that affect height [7].

Rare variants can in principle be identified in family-based linkage studies with lower sample size requirements than association studies. 21 family-based studies of height have been conducted, mostly prior to the GWAS era [12–32]. Only a few of these studies detected Quantitative Trait Loci (QTLs) linked to height at the accepted level of genome-wide statistical significance, with the three most convincing findings all reported by a single study with the largest sample size of subjects from one ethnicity [23]. Moreover, few if any of the reported QTLs were replicated across multiple studies [14,23,32].

We sought to overcome some of the limitations of previous linkage studies by focusing on several factors that influence statistical power, including the minor allele frequency (MAF) of causal variants, the strength of linkage between causal and typed variants, and the quality of genotyping and phenotyping [33,34]. Specifically, we attempted to increase power by studying the genetic architecture of height in very large nuclear families (10 to 20 siblings per family) from a founder (Jewish) population. We acquired highly accurate measurements of height, and we used dense genotyping arrays to fully reconstruct the inheritance patterns in these families. The use of large pedigrees drawn from a population with a small effective population size should increase the effective allele frequency of variants of interest, enabling their detection in a study with a modest sample size.

## Results

### Increasing effective allele frequencies by studying very large nuclear families

To increase the power to detect effects of rare variants, we sought to increase their effective frequency in our cohort by studying very large nuclear families. A rare variant carried by a parent of such a family automatically rises to a frequency of ~25% among the children. However, this effect is rapidly diluted when many unrelated small families are combined, as was done in all previous family studies of height. To minimize such dilution, we recruited 397 participants from 29 very large nuclear families containing 10 to 20 siblings per family (mean = 12 siblings). In addition, siblings in eight of the nuclear families have first cousins in several other nuclear families in the cohort. Any variant segregating in our cohort has a minimum expected MAF of ~1% if it is present in only one family, and higher if it is present in multiple families, regardless of its frequency in the general population. See **S1 Table** for more details on the cohort.

### Increasing allele frequencies by studying a founder population

Another approach to increase MAF is to study a founder population, where some variants that are rare in a cosmopolitan population can rise to high frequencies due to a small number of founders and subsequent genetic drift. We therefore recruited our cohort from the Jewish population. Specifically, 80% of our cohort consists of Ashkenazi Jews (AJ). Today’s 10 million AJ have an effective population size (N_e_) of ~350 as a result of a founder event ~700 years ago [35]. This effective population size is small even compared to other founder populations such as Finland (N_e_≈3000) [36] and Iceland (N_e_≈5000) [37]. Two unrelated AJ on average share ~30 times more of their genome in long (>3Mbps) identity by descent (IBD) segments than two unrelated non-Jewish Caucasians [38]. As a result, variants that are rare in other populations can rise to high frequencies in AJ. Indeed, at least 40 Mendelian genetic diseases in AJ are caused predominantly by such founder mutations that are non-existent or rare in other populations [39,40]. The other 20% of our sample are Jews of other ethnicities. Previous studies showed that Jews who are not Ashkenazi are on average closer genetically to Ashkenazi Jews than to their non-Jewish neighbors [41].

**Fig 1:**
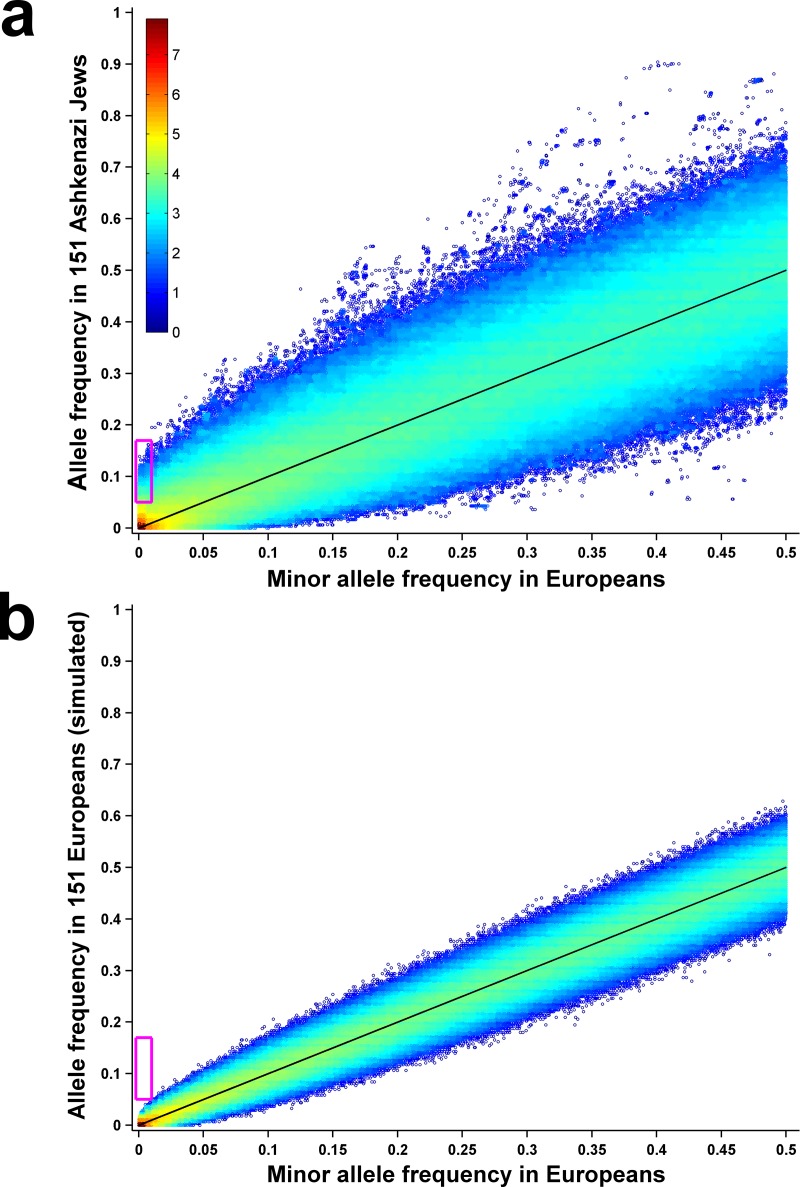
Founder effect in Ashkenazi Jews. (**a**) Minor allele frequency of variants in 7509 Europeans (X-axis) vs. their allele frequency in 151 Ashkenazi Jews (Y-axis). Each dot represents one of ~90.5 million genetic variants from the gnomAD database. Color displays density, i.e. the number of variants in that region of the plot, on a log10 scale. The magenta rectangle encloses all variants that are rare (MAF<1%) in Europeans but common (MAF≥5%) in Ashkenazi Jews. Black line shows y = x. (**b**) Same as in (a), but with allele frequency in a random sub-sample of 151 Europeans shown on the Y-axis.

To estimate the shift in allele frequencies between the European and AJ populations, we compared allele frequencies in whole genomes of 7509 non-Finnish Europeans and 151 Ashkenazi Jews, both from the gnomAD database [42] (**Fig 1**). The database contains 90.5 million bi-allelic high quality variants found in Europeans, and 82.5 million (91%) of these are rare (MAF<1%). 95% of the rare variants found in Europeans are not observed in the AJ sample, but 33,331 variants that are rare or not observed in Europeans are common in AJ (allele frequency ≥5%), and 757 of them reach allele frequency ≥10%. To test whether the increase in the frequency of rare variants in AJ is a consequence of small sample size, we used the European allele frequencies as probabilities for randomly sampling 151 Europeans from gnomAD and repeated the analysis. No rare variant reached a frequency of ≥5% in this sub-sample (**Fig 1B**).

Increasing the MAF of a variant increases the power to detect that variant in several ways. First, power is dependent directly on MAF. Second, rare variants tend to have a lower quality of genotyping and imputation [33], and increasing MAF allows for a higher quality of both, especially when using a population-matched reference panel for imputation. Third, the effect sizes of variants are typically inversely related to their frequencies in cosmopolitan populations [6,7], and therefore causal variants that are rare elsewhere but are common in our study population may have larger effect sizes. The combination of these factors, together with our use of large families and accurate phenotyping (see below), has the potential to increase power to detect Quantitative Trait Loci (QTL) that influence height.

### Phenotype accuracy

Most previous height studies used measurements acquired incidentally as part of studying other traits or diseases. Such measurements may suffer from low accuracy. To ensure phenotype accuracy and increase statistical power, we measured the height of each participant to the nearest 0.1 cm, with four technical repeats, and with one researcher (D.Ze.) conducting all measurements and using the same measurement system. We tested different methods to correct heights for age and sex and used a non-linear correction for age that maximized heritability in our sample.

### Multiple siblings and dense SNP arrays enable reconstruction of fully informative inheritance patterns for linkage analysis

Nearly all previous family linkage studies of height [12–32] used sparse maps of microsatellite markers, which provide incomplete inheritance information content and a corresponding reduction in statistical power to detect QTL [43]. A dense map of single nucleotide polymorphisms (SNPs), combined with multipoint linkage analysis, can generate near-perfect information content [43], but existing computational tools are not well-suited to carry out multipoint analysis with hundreds of thousands of markers in very large families. To obtain fully informative inheritance patterns at every position in the genome, we genotyped ~630,000 SNPs and developed a method for identity-by-descent (IBD) reconstruction that leverages information from the large number of siblings in each family. Briefly, we compared genotypes of each sibling pair to identify IBD segments, and then used these segments identified across all pairs to reconstruct, at every genomic position, the fully informative inheritance pattern of all four parental haplotypes in the children (**S1 Fig**). Every recombination event in a sibling was identified as a change in his or her IBD relationship pattern with the other siblings. Because the number of recombination events per chromosome per participant (typically 1–2) is much smaller than the number of siblings in each of our families, we were able to identify these IBD switching events with high certainty, even in the absence of parental genotypes. To test the accuracy of the method, we calculated the average pair-wise IBD sharing for the genome. Two siblings shared zero, one or two alleles IBD for 24.9%, 50% and 25.1% of the genome, compared to the theoretical expectation of 25%, 50% and 25%. The IBD segments identified by our method provide near-perfect inheritance information for linkage analysis between genomic loci and height.

### QTL mapping

To identify regions of the genome that co-segregate with height differences (QTLs), we conducted linkage analysis by contrast tests (ref. [44]). Briefly, at every position in the genome, we compared the heights of siblings that inherited one of the two possible haplotypes from a given parent to the heights of those who inherited the other haplotype. We used permutations of height among siblings in a family to calculate significance, which we express as an equivalent LOD score (**S2 Fig**). It has been shown [45] that for sib pair analysis, genome-wide significance at an FDR of 5% corresponds to LOD≥3.6 (equivalent to p-value ≤ 2X10^-5^). We identified two significant QTLs, on chr14:71,145,000–71,855,000 bp (hg19 genome coordinates) with LOD = 4.13, and on chr17:78,845,000–79,695,000 bp with LOD = 3.8. It is important to note that our QTL sizes are large, spanning hundreds of thousands of base pairs (as is typical in linkage analysis), compared to GWAS results that identify variants in LD blocks that have typical sizes of tens of thousands base pairs, and that sometimes can even indicate the causal SNPs if they are genotyped. Permutation analysis showed that 0.11 QTLs are expected by chance at this threshold, which corresponds to an empirical FDR of 5.5%, in close agreement with the theoretical value.

The QTL on chromosome 14 includes two genes: PCNX, an oncogene [46] and homolog to a drosophila component of the Notch signaling pathway [47], which functions in several developmental processes, and MAP3K9, a Mitogen-activated protein kinase. The QTL on chromosome 17 contains 25 genes, including an interesting candidate, RPTOR—a binding Partner of Target of Rapamycin (TOR) which control cell growth [48]. Mutations in RPTOR have been shown to cause significant reduction in body size in flies [49] and mice [50].

**Fig 2:**
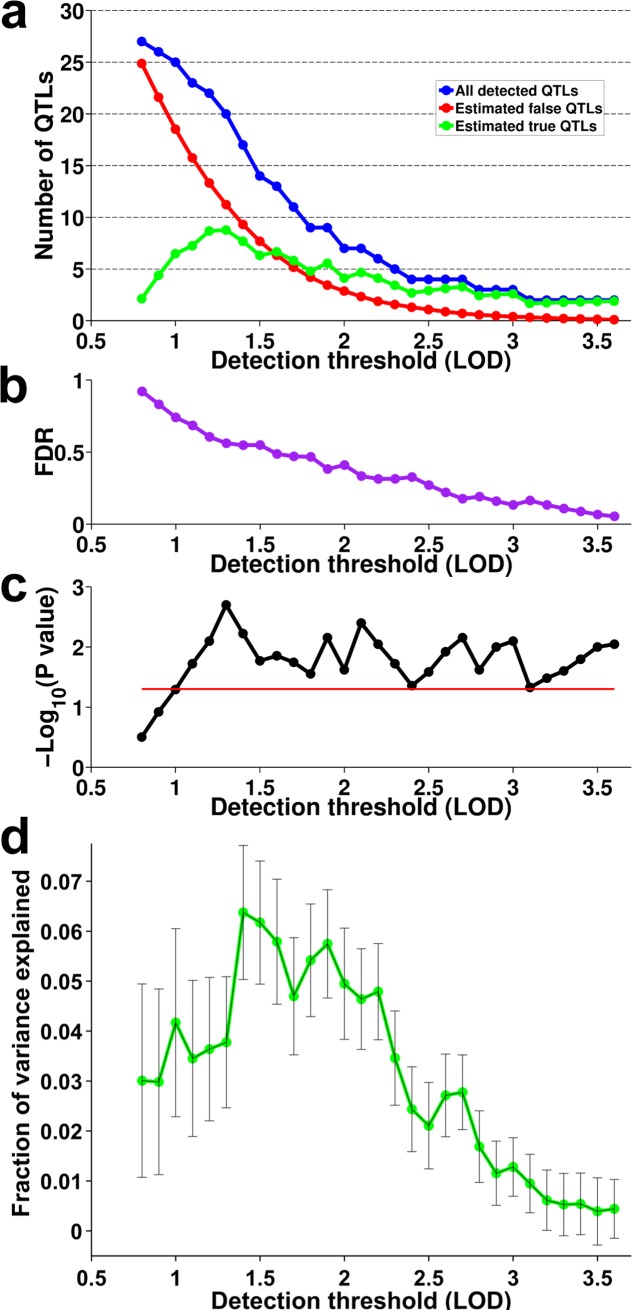
QTL mapping. (**a**) For each LOD detection threshold, the number of detected QTLs is plotted in blue, the expected number of false-positive QTLs based on permutation analysis is plotted in red, and the difference between these two numbers is plotted in green. (**b**) Permutation-based false discovery rate at each detection threshold (**c**) Statistical significance of the number of detected QTLs at each threshold, shown as–Log10(permutation-based P-value). Red line shows P = 0.05. (**d**) Variance explained by QTLs in a cross-validation framework, compared to a null model with random genomic segments of similar size. Error bars represent the standard errors (SE) of the median of the variance explained in the 100 training/test sets.

We reasoned that because height is a complex trait that is governed by multiple loci with relatively small effects, an FDR analysis of larger sets of loci with lower locus-specific significance would be appropriate and could increase the sensitivity of our study. This approach has been previously employed in GWAS [51], but was not attempted in previous linkage studies of height. To carry out such analysis, we compared the number of observed QTLs for a range of LOD score thresholds below 3.6 to the number expected in the absence of true signals, determined from permutations. As expected, lower thresholds resulted in larger numbers of detected QTLs, with an increasing FDR (**Fig 2A and 2B**). Importantly, the total number of detected QTLs remained significant (P<0.05 compared to permutations, **Fig 2C**) from the strictest threshold (LOD = 3.6; 2 QTLs; P = 0.009; FDR = 5.5%), down to LOD = 1.1 (24 QTLs; P = 0.019; FDR = 68%; **S2 Table**). We used the FDR to estimate the number of true QTLs as a function of decreasing detection thresholds (**Fig 2A**). The number of true QTLs maximizes at LOD = 1.3, where we estimate 8.8 true QTLs out of 20 that were detected (P = 0.002; FDR = 56%).

### Variance explained by QTLs

To estimate how much phenotypic variance can be explained by the detected QTLs, and to test whether the detected loci tag common SNPs identified by previous height GWAS [52], we conducted variance partitioning in a cross validation framework designed to avoid potential overfitting that could result from detecting QTLs and estimating their effect sizes in the same dataset. We generated 100 training sets that each randomly sampled 2/3 of the families. In each set, we mapped QTLs as described above. The results were similar to those obtained with the full dataset (**S3 Fig**), although on average, fewer QTLs were in the training sets as a consequence of the smaller sample size. We then used the 1/3 of the families held out of each training set as a test set, and simultaneously estimated the contributions of three different sources of genetic variance to height variance in a variance components model (implemented in the software package GCTA [53]). Specifically, the model included variance component terms for the detected QTLs (using SNPs from the detected QTLs in the training sets), the common SNPs associated with height by GWAS [52], and the overall genomic relatedness among individuals; the latter controls for pedigree structure, and can also capture a polygenic signal of height. To further control for upward bias in the estimation of variance explained in the test set due to shared environment of family members and tagging of polygenic background, we used as a baseline the variance explained in GCTA in a similar model with the same number of SNPs but from random genomic segments similar in size to the real QTLs (“random QTLs”). The reported variance explained by QTLs (**Fig 2D**) are after subtraction of variance explained by the “random QTLs” in the null model.

The total variance explained by the QTLs increased as the detection threshold was lowered and a larger number of QTLs were included in the model, but the variance explained over and above the null model initially also increased (**Fig 2D**). For example, at LOD≥3, we detected an average of 0.74 estimated true QTLs per training set (1 QTL at FDR = 26%), and these explained an average of 1.3% of the phenotypic variance above the null model in the test sets. At a lower detection threshold of LOD = 1.9, we estimated an average of 3.7 true QTLs (7 QTLs at FDR = 47.5%), and these explained on average 5.8% of the variance above the null model. For low detection thresholds (LOD≤1.3), the variance explained above the null model decreased, presumably because too many false discoveries were included.

To test whether QTLs explained variance in height by tagging previously discovered height-associated common variants, we ran the variance components model with and without including the GWAS SNPs. The variance explained by the QTLs in both models was similar (0.1% difference, t-test P = 0.72, **S4 and S5 Figs**). This result suggests that the QTLs we identified are novel and are not simply tagging common SNPs that were previously identified by GWAS. The GWAS SNPs explain in our variance components model 11.2±1.8%, uniformly across LOD thresholds regardless of the number of QTLs they compete against. This is similar to the amount of variance explained reported in the study which originally identified these GWAS variants ^3^.

### Specific chromosomes contribute disproportionally to height and in accordance with the QTL signals

**Fig 3:**
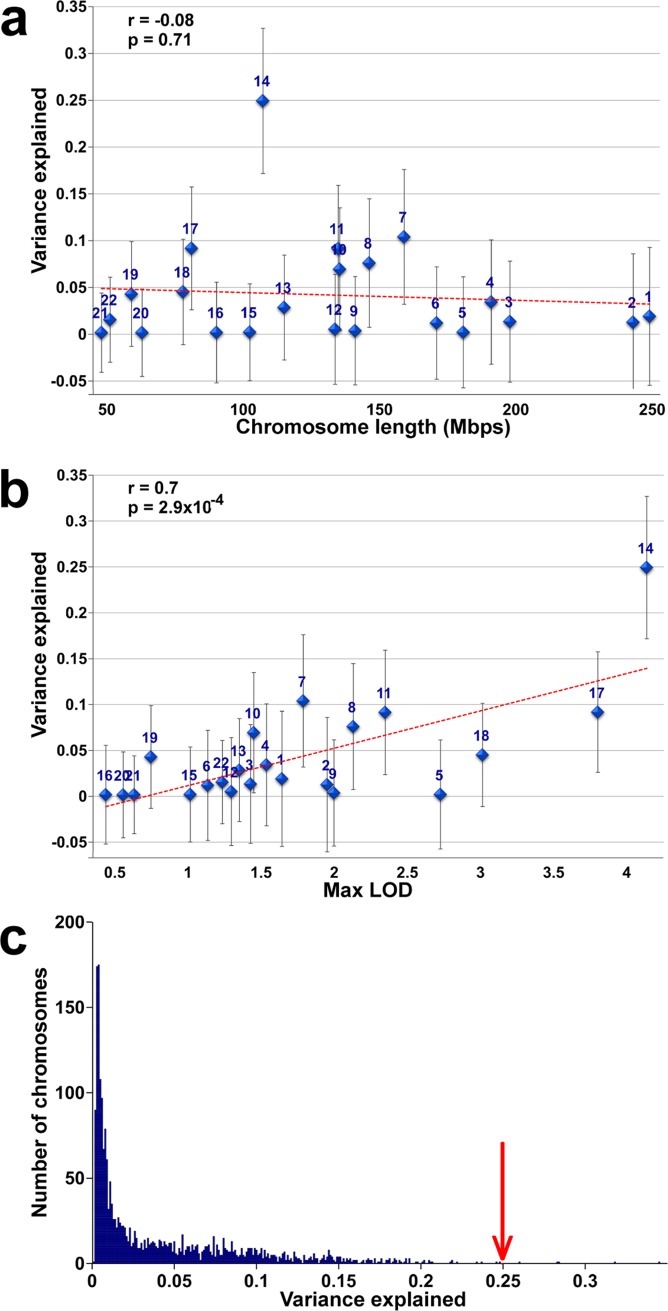
Height variance explained by chromosomes. (**a**) Height variance explained by each chromosome ± SE is plotted against chromosome length. Red line shows the linear fit. (**b**) Height variance explained by each chromosome ± SE is plotted against the maximum LOD score on that chromosome. (**c**) Histogram of variance explained by each of 2200 chromosomes simulated under an infinitesimal model. Red arrow shows variance explained by chromosome 14 in the real data.

To assess how much phenotypic variance can be explained by entire chromosomes, we estimated a genomic relatedness matrix (GRM) from all the SNPs on each autosome, and let all 22 GRMs compete together at the same time in a variance components model to explain phenotypic variance (**Fig 3**). We found no correlation between variance explained and chromosome length (Pearson r = –0.08, P = 0.71), although we cannot rule out that such a correlation exists, as the standard errors in the estimation of single chromosome contributions are large. In contrast, the variance explained by chromosomes is highly correlated with the top LOD score on each chromosome (Pearson r = 0.7, P = 2.9x10^-4^; Spearman r = 0.6420, P = 0.0016). This correlation arises in part from the results for chromosome 14, which explains the most variance in the variance components model (25%±7.8%) and has the single most significant QTL. To test whether the correlation is driven solely by chromosome 14, we omitted it from the analysis. The correlation fell but remained significant (Pearson r = 0.53, P = 0.015; Spearman r = 0.59, P = 0.006).

These results suggest that at least in our sample, variance explained by some of the chromosomes captures contributions of small regions with large effects rather than solely infinitesimal contributions distributed throughout the entire chromosomes. To investigate this further, we simulated 100 sets of phenotypes from an infinitesimal model, in which normally distributed small effect sizes were randomly assigned to all SNPs in the genome while maintaining the overall heritability. For each simulated data set, we calculated the distribution of variance explained by entire chromosomes. The correlation between variance explained and chromosome length in the different simulated sets was r = 0.19±0.19 (Mean±SD), stronger than the r = –0.08 observed for the real data, although the difference was not statistically significant (P = 0.09, **S6 Fig**). The observed variance explained by chromosome 14 was significantly higher than expected from the infinitesimal model. Only 5/2200 chromosomes in the simulated data sets explained as much as 25% of the variance (Bonferroni corrected P = 0.05, **Fig 3C**), and all of these five observations were for chromosomes that are longer than chromosome 14.

## Discussion

Here, we studied height in a unique cohort of very large nuclear families from a founder population. This strategy was designed to increase the effective allele frequency of some variants that are otherwise rare, thereby also increasing our power to detect their effects on height. This approach enabled us to detect significant QTLs for height in a study with modest sample size. We also used FDR analysis to identify a larger number of QTLs that were highly significant as a set, despite few of the QTLs achieving significance individually. Using a variance components model, we showed that these QTLs explained 6% of the variance in height in a cross validation framework, and that they were not tagging common variants previously identified as associated with height by GWAS. The actual fraction of variance explained by the QTLs is likely higher because of the conservative nature of the estimation procedure. Further, we showed that the variance contributed by chromosome 14, and possibly by some of the other chromosomes, arises at least in part from small regions with large effects (which correspond to the detected QTLs), rather than solely from infinitesimal contributions distributed throughout the entire chromosomes.

Taken together, these results suggest that variation in height in our sample arises from a combination of a small number of QTLs with large effects and a large number of common variants with small effects. Because the detected QTLs are not tagging previously identified common variants, they likely arise from variants that are elevated in frequency in the AJ population. Although we have not identified the specific variants underlying the QTLs, we speculate that candidate variants can be identified by sequencing the parents of the pedigrees and searching for variants that are rare in other populations, common in AJ, and follow segregation patterns consistent with the QTL signals. The approach described in this paper, coupled with recruitment of additional large families (which are abundant in the Jewish population [54,55]), may provide further insights into the genetic basis of height and the role of population-specific vs. cosmopolitan variants, and may serve as a complement to GWAS for genetic investigations of other complex traits.

## Methods

### Ethics statement

This study was approved by the IRB of Shaare Zedek Medical Center, Jerusalem, Israel (IRB#131/12), Princeton University, Princeton, NJ, USA (IRB#0000006027), and UCLA, Los Angeles, CA, USA (IRB#14–000357). In all cases written consent was obtained.

### Participants recruitment and measurements

Participants were recruited in Israel and in the US after receiving IRB approvals in both locations. All participants gave written informed consent, then filled a questionnaire about their growth process, medical history, lifestyle during growth years (nutrition, sleep, physical activity, etc.), and ancestry origins and heights. Participants’ heights were measured with a Seca 213 mobile measurement system to the nearest 0.1 cm with 4 repeats (participants stepped off and on the measurement system for repeated measurements). We also measured sitting height (±0.1 cm, 3 repeats) and arm span (±1 cm, 3 repeats). The participants donated saliva samples into DNA Genotek OGR-500 tubes from which DNA was extracted by Ethanol precipitation. The samples were genotyped by RUDCR (Rutgers) using Affymetrix Axiom Biobank Array (~630,000 SNPs).

The Heights distributions in our sample were normal with 210 males at 172.7cm ± 5.7cm (Mean ± S.D.) and 187 females at 161.6 ± 5.5 cm (Mean ± S.D.) and are representative of the Israeli population (Mean = 173.7 cm for Israeli males and 160.3 cm for females [56]). See **S1 Table** for more details on the cohort.

### Height correction for age

Longitudinal studies have shown that people “shrink” non-linearly with age, and that the rates of shrinkage also differ for men and for women [57,58]. We therefore used the data of a previous longitudinal study [57] to derive sex specific non-linear equations for height shrinkage as a function of age. Their data included measurements of the rate of height change for every decade for men and women separately from age 20 to 90. They showed that men and women start shrinking around age 30 and that between age 30 and 80 the rate of height loss increases linearly with age (i.e. constant acceleration of height loss). We plotted these rates and used a linear fit to derive the dependence of the rate of height loss on age. Height loss rate increases by 0.00416 cm per year for men, and by 0.00641 cm per year for women both starting at age 30. We integrated these equations to receive quadratic formulas for height loss:

Defining:

H≡HeightLoss

For males:

From plotting longitudinal data [57] and fitting a linear regression:

$$\frac{dH}{dAge}=-0.00416\times Age+0.124$$

Age when starting to shrink:

$$\frac{dH}{dAge}=0\to {Age}_{start\;shrinking}=\frac{0.124}{0.00416}=29.8$$

Defining *t* ≡ *Age* − 29.8

The rate of height loss after age 29.8:

$$\frac{dH}{d(t)}=-0.00416\times t$$

Integrating to get the height loss as a function of time (after age 29.8):

$$H(t)=\int \frac{dH}{d(t)}=\int -0.00416\times t=-0.00208\times t^{2}+C$$

H(t=0)=0→C=0

$$H(t)=-0.00208\times t^{2}$$

For females:

$$\frac{dH}{dAge}=-0.00641\times Age+0.18975$$

$${Age}_{start\;shrinking}=29.6$$

t≡Age−29.6

$$H(t)=-0.003205\times t^{2}$$

Finally, since participants reported ages in full years, we approximate:

HeightLoss(Age)={0,Age≤30−0.00208×(Age−30)2,Males,Age>30−0.003205×(Age−30)2,Females,Age>30

To compare our quadratic correction for age to a linear one, we first estimated the linear correction from our data by using SOLAR [59] and applying age and sex as covariates in a linear model. The linear correction for height was 0.0995*(Age-35.084) + Height corrected for sex. We then compared the linear and quadratic corrections by estimating (using SOLAR) the heritability of our cohort for heights corrected by the two models. The heritability of height after a quadratic correction to age was h^2^±S.E = 0.86±0.07, higher than the h^2^±S.E = 0.81±0.08 achieved after a linear correction. This improvement of the quadratic model over the linear one might be an underestimation since the quadratic correction was estimated from a different cohort of a longitudinal study, while the linear correction was estimated with the same data that was used to estimate the heritability. For any further analysis we therefore used the quadratic correction.

### Height correction for sex

To correct height for sex we standardized (z score) the heights of females and males separately and then pooled the standardized heights together.

### Power analysis

To calculate power, we used ANNOVA F-test, as follows in Appendix A5 of [34]:

The non-centrality parameter (NCP):

$$\lambda =\frac{f*n*(MAF-{MAF}^{2})*\beta^{2}}{1-0.5*[h^{2}-2*(MAF-{MAF}^{2})*\beta^{2})}$$

where:

f = number of families, n = number of siblings per family, MAF = Minor Allele Frequency, β = effect size (assumed 1 SD), h^2^ = heritability (assumed 80%) and:

$$Power=Pr[F_{{df}_{1},{df}_{2},\lambda }>F_{{df}_{1},{df}_{2},[1-\alpha ]}]$$

Where df1 = 2*f, df2 = f*n-3, α = significance level corresponding to the LOD detection threshold

In R:

chi_threshold = lod_threshold * 2 * log(10)

a = pchisq(chi_threshold,1,lower.tail = FALSE)

power = pf(a,df1,df2,ncp,lower.tail = FALSE)

### Quality control of genotyping

The Affymetrix Axiom Biobank Array was used for genotyping. It covers 628,679 SNPs distributed across the genome. Samples had on average 8043 ± 3291 (mean ± S.D) SNPs reported as “No Call” (1.3% ± 0.52%), and 48,787 ± 12881 SNP calls (7.8% ± 2%) reported as “Low Quality” (P>0). To assess the experimental technical error, we genotyped one sample 4 times (in different Axiom array 96 plates). For the 6 two-way comparisons, excluding all “No Calls” in each compared pair, we had an error rate of 9.1x10^-3^ ± 2.5x10^-3^ (mean ± S.D) i.e. 1 SNP in every ~110 SNP is called wrongly. However, most of these errors were in the “Low Quality” category, since while only ~8% of the calls had low quality scores, when they were dropped from the comparisons, the error rate dropped ~20 fold to 4.45 x10^-4^ ± 2.7x10^-4^ or 1 SNP called with an error in every ~2247 SNPs. Given these results, we did not use any SNP with a low quality call score for IBD calling.

### IBD reconstruction

To reconstruct the inheritance patterns in each family, we used only the informative SNPs that are not homozygote identical in all siblings of the family. Typically, it reduced the number of useable SNPs in a family from ~630,000 to ~180,000 SNPs, reflecting the high level of genetic homogeneity in our population. To increase accuracy, we excluded any low quality SNP calls (Axiom Biobank array calls with a “Confidence” value > 0 or a “No Call”, a total of approximately 4% of the SNPs of each participant). This exclusion left ~150,000 informative SNPs for comparison between each sib pair. For each sib pair, we compared the SNP calls along the genome to partition the genome into regions with opposite homozygous calls (indicating a region with 0 shared alleles), regions with no opposite homozygous calls but heterozygote calls in one sib vs homozygote calls in the other (indicating 1 shared allele) and regions with only identical homozygote calls (2 shared alleles). To avoid false positives due to genotyping errors we required a stretch of at least 3 SNPs of the same type (opposite homozygotes, one heterozygote vs one homozygote, or two identical homozygotes) within 2Mbps, 1Mbps and 1Mbps accordingly, to declare a region as 0, 1 or 2 alleles shared. (See **S1 Fig** for an example). To correct for errors of IBD calls between each sib pair we used the multiple siblings’ information by comparing all the sib pairs IBD within a family. For example, if sib #1 and sib #2 (1–2) share 2 alleles in some region and so do sib pairs 1–3, 1–4, 2–3 and 2–4, we would expect sibs 3–4 to also share 2 alleles in this region and therefore a 1 shared allele for this sib pair would be likely a false negative and we should correct it to 2 shared alleles. We corrected such contradictions by applying the minimal number of corrections to sib pairs IBD calls while prohibiting creating any new contradictions with other sib pairs by the correction.

Examining the matrix of the shared allele numbers between all sibling pairs shows the siblings falling into 4 groups (4 possible values for lines in the matrix) or less, as expected by the 4 possible combinations of grandparental alleles. Advancing along a chromosome, these matrices remain identical up to some chromosomal position where one of the siblings changes suddenly its allele shared numbers with all other siblings and move from one grandparental allele group into another, indicating a recombination event. Further, the new grandparental alleles combination that the sibling switch into allows us to infer in which of his two haplotypes the recombination event occurred. An example of a fully phased IBD reconstructed map of a nuclear family is in **S1 Fig**.

### QTL mapping

We conducted QTL mapping by marker contrast tests ([44], chapter 16). We divided the siblings of each family every 5000 bases along the genome into two groups according to which grandparental haplotype they inherited from a specific parent (the IBD reconstruction). We performed simple linear regression and calculated the coefficient of determination (R^2^) between the two groups and the siblings’ heights. We repeated the calculation for the two haplotypes inherited from the other parent at the same genomic position to get a total of two R^2^ scores (one from each parent) that correspond to the association of this position to height differences (See example at **S1 Fig**).

To adjust for the different number of haplotypes underlying the two correlations in different genomic positions, we conducted permutation analysis. We permuted randomly the heights of the siblings within each family 1000 times while keeping the genetics fixed and calculated the resulting R^2^ scores for each genomic position. We then calculated two local P values for each genomic position for each family as the number of times a similar or larger R^2^ was achieved in the permuted families compared to the real family. This procedure also normalizes the signal for family size. We then take the lower of the two single family local P values, which allows for capturing also dominance effects (results however were robust to taking the average) and combine the signals from all families by taking the average of these local P values over all the families at each position. We then repeat the same calculation for the 1000 height permuted sets of families, and calculate an empirical genome wide P value by counting how many times each local P value or lower from the real families appears anywhere in the genome in any of the height permuted families’ combinations. Lastly, to make it easier to compare to previous linkage studies, we transform the global genome wide P values into LOD scores by using the inverse of the Chi-Square cumulative distribution function, as described in [60]. The LOD scores for all chromosomes are plotted in **S2 Fig**.

### Determining separation between QTLs

To estimate the genomic distance in which two nearby QTLs can be called as independent, we investigated how far along a chromosome a LOD score is still correlated to other LOD scores of nearby positions. We calculated the absolute difference of LOD scores between any two positions on any given chromosome, and averaged over all chromosomes to get the mean absolute difference of LOD scores as a function of genomic distance (**S7 Fig**). As expected, close genomic regions show, on average, similar LOD scores (due to linkage), and increasingly larger genomic distances show monotonically increasing absolute LOD differences. This holds true up to a genomic distance of ~33Mbps where the LOD score difference reaches its median level, and for larger distances the LOD score absolute difference fluctuates around its median value. We infer that association between genotype and phenotype at some genomic position has on average no influence through linkage over the association at a distance larger than 33Mbps. We therefore count regions with LOD scores above detection threshold as belonging to the same QTL if they are less than 33Mbps apart.

### Significance testing of QTLs

A Quantitative Trait Locus (QTL) is called for every linkage signal peak that is above some LOD threshold. To avoid noise and linkage over large distances translating into multiple peaks we count peaks that are less than 33Mbps apart as one QTL. We define the QTL confidence interval as the region between the furthest positions from the QTL peak that are above 1 LOD drop from the peak’s LOD score and that are no further than ±33Mbps from the peak’s position.

To calculate the empirical P value and false discovery rate (FDR) for the total number of QTLs, we use permutation analysis. We repeat the QTL detection procedure for the 1000 combinations of height permuted families and calculate the distribution of the number of QTLs that are expected by chance anywhere in the genome. We then compare the total number of QTLs detected for the real families to the distribution of the number of QTLs detected in the permutation analysis. We calculate the significance (P value) of the real families’ QTLs as the fraction of permutations that yield an equal or larger number of QTLs than the real families, and the FDR as the ratio between the average QTL number per permutation (the expected number of QTLs by chance) and the number of QTLs from the real families (the observed number of QTLs). We define the estimated total number of true QTLs as the difference between the observed and expected QTL numbers.

The required detection threshold to call linkage as genome-wide significant with a low false positive rate of 5% (LOD score = 3.6 for sibling pairs, or an equivalent P value = 2X10^-5^) was established over two decades ago [45] and has been the gold standard for family studies. Using this strict threshold is appropriate for Mendelian traits where one expects only a single locus to influence the trait and given the high costs of perusing and fine mapping QTLs to find the causal genetic variant. Height however is a complex trait and is most probably governed by multiple loci with smaller effects. We therefore increased the power to detect QTLs by investigating the space of lower detection thresholds. We start from the traditional LOD = 3.6 threshold and gradually lower the detection threshold at constant increments of 0.1 LOD, thus increasing the sensitivity and detecting more QTLs at lower thresholds. Individually, QTLs that are identified below LOD = 3.6 might not be significant, but as a group, the total number of detected QTLs can be significant when compared to the total number of QTLs detected in the permutations. The increased sensitivity to identify more QTLs comes at the expense of reduced specificity (higher FDR), i.e. more QTLs are detected at lower detection thresholds, but a larger fraction of these QTLs are false (and it is not possible to know which specific QTLs are the false ones). However, as long as true positives accumulate faster than false positives, the absolute number of true QTLs that are identified increases.

See **Fig 2** for the P values and FDR for the total number of detected QTLs for different detection thresholds. See **S2 Table** for a list of all detected QTLs. All genomic coordinates refer to genome build hg19.

### Haplotype phasing

To correct genotyping and IBD inference errors, and to facilitate imputation, we phase the genotypes of all participants into haplotypes. We first use the phased IBD structure that we reconstructed in a previous step. For each SNP we compare the SNP calls of the children that belong to the four different possible combinations of grandparental haplotypes. If the siblings in one of these groups are homozygote for this SNP, both haplotypes are assigned with the called allele. If they are heterozygous, we examine the SNP calls for the two other groups that share with the group in question exactly one allele IBD.

In the cases where heterozygote calls cannot be phased in the above manner (e.g. when all siblings are heterozygotes) we use information from the parents (if existing in the data). First, for each chromosome we compare the haplotypes in the already solved positions to the SNP calls of the parents in order to identify which of the parents is carrying haplotypes 1 & 2 and which carries haplotypes 3 & 4 along the entire chromosome. We then use homozygous SNPs of the parents to phase heterozygous SNPs of the children.

Using only the IBD we were able to phase ~95% of the SNPs. Unphased SNPs included SNPs where not enough siblings or parents had good quality genotype calls, SNPs where no informative homozygote participant existed or SNPs where phasing by different siblings or parents contradicted. To phase the remaining SNPs we used the phased haplotypes of the entire study population as reference. For each unphased SNP within a specific family, we use all the previously solved haplotypes in other families that include this SNP. We preferably use the longest haplotype that matches on both sides of the unknown call, and when there are contradicting cases we use the call that matches the majority of the solved haplotypes. When there is no usable information (<0.1% of cases) the call is determined to be the B allele (due to the Affymetrix definition of the A and B allele on their chip).

### Quality control of IBD reconstruction and haplotype phasing

To test the accuracy of our IBD reconstruction and haplotype phasing we compared the raw genotype calls as read from the Affymetrix Axiom Biobank array, to the genotype calls that can be inferred back from the calculated haplotypes. Since the reconstructed IBD is used for phasing the haplotypes, mismatches between the experimentally read genotypes and the inferred genotypes can be caused either by experimental errors in genotyping, or by analysis errors in the IBD reconstruction or haplotype phasing. We observed two types of mismatch patterns. First, blocks of continuous mismatches between the observed and inferred genotypes, that are immediately adjacent to inferred recombination positions. Such blocks most likely indicate errors in the IBD calling due to errors in identification of the exact recombination position. The rate of this type of error was low, as blocks of mismatches covered <1% of all chromosomes of all participants. We reduced it further by correcting manually IBD calls that created mismatch blocks larger than 1Mbps and then iterated the haplotype phasing pipeline and the above quality control. The second type of errors is sparsely distributed mismatches. Since our nuclear families are large and typically at least several siblings share at each genomic position the same two grandparental haplotypes from their parents, if our IBD reconstruction is correct, the inferred genotypes can be in theory more accurate than the experimentally measured ones since experimental errors in one sibling could be corrected by the other siblings that share the same two grandparental haplotypes, or by other families who share a haplotype in this genomic region. When experimentally low quality SNP calls (Confidence value > 0 in the Affymetrix array reading) were included, the frequency of SNP calls differing between the experimentally measured and analytically inferred for the 397 participants was 9.1x10^-3^ ± 2.5x10^-3^ (mean ± S.D), practically identical to the experimental error rate measured as mismatches between technical repeats. This suggests that most SNPs with experimental genotyping errors due to low quality are indeed corrected by the multiple siblings and parents IBD information. When low quality genotype calls are excluded from the comparison, the mismatch rate drops ~8 fold to 1.2x10^-3^ ± 4.9x10^-4^ (mean ± S.D), i.e. from 1 error in every ~110 SNPs to 1 in every ~866 SNPs. The total number of mismatches that we observe between experimentally genotypes and analytically inferred genotypes is on average ~726 errors per sample for the 628,456 SNPs on the array (excluding the Y chromosome and mitochondria), of which ~280 errors are expected to be errors coming from experimentally determining genotypes (based on the expectation of an experimental error of 1 in every ~2247 SNPs), and ~446 errors are expected to come from errors in the IBD reconstruction and haplotype phasing pipeline. Overall these quality control calculations suggest a low error rate of 446/628,456 = 7.1x10^-4^ for our IBD reconstruction and haplotype phasing pipeline (1 in every 1409 SNPs), while correcting most of the individual sample genotyping experimental errors and SNPs that were not called. This is important as the inferred genotyped and not the experimentally measured genotypes are used for the downstream analysis of variance partitioning and predictions of height.

### Estimation of the variance explained by the QTLs

To assess how much phenotypic variance can be explained by the QTLs, we conducted variance partitioning in a cross validation framework. We divided randomly our data into 100 training and corresponding test sets. Each training set contained 19 families and the corresponding test set the other 10 families. We constrained the random choice of families so that each training set contained two thirds of all participants and each test set one third of all participants (±2 participants).

We conducted linkage analysis as detailed above on each training set and identified QTLs. We used only the autosomal QTLs for the next steps.

We took the coordinates of the QTLs into a corresponding test set, and from the genotypes of the test set participants took 100 SNPs closest to each QTL peak (~1Mbps per QTL) and concatenated these SNPs together (e.g. for 7 QTLs we took the genotype calls of 700 SNPs). We chose to use 100 SNPs since using less SNPs explained less variance, probably since there are not enough SNPs to capture the exact genetic relatedness between participants in these regions (e.g. two participants can be identical by state (IBS) for several SNPs, while not identical by descent (IBD), for example if they are both homozygote to a common variant with high frequency). Increasing the GRMs to include more than 100 SNPs per QTL did not result in explaining more variance, suggesting that the genetic variance relevant to the trait is captured in the 100 SNPs (~1Mbps) around a QTL.

We used the SNPs in the QTLs as input to the software GCTA [53] to create a Genomic Relatedness Matrix (GRM), representing the genetic similarity between all participants in the test set for the specific coordinates of the QTLs (hence the QTL GRM). To control for pedigree structure, the overall genetic similarity between participants and shared environments, we built a second GRM based on all the genotyped SNPs of the genome (~350,000 informative SNPs, hence whole genome GRM). To control for QTLs explaining variance through tagging of known common variants for height, we constructed a third GRM from 650 GWAS [3] known common variants that we could genotype or impute (hence GWAS GRM). We fit a joint variance component model, where variance in height is the sum of the random effects from GRMs (covariance structures) built from QTLs, the whole genome, and GWAS snps, as described above using GCTA [53], with the EM algorithm. Since the QTLs GRM might explain some phenotypic variance simply because is correlated to the whole genome GRM and the GWAS GRM, we repeated the analysis for each test set 100 times, but with a random QTLs GRM made from random regions in the genome that have equal number (100) of SNPs as in the real QTLs GRM. We constrained these randomly chosen regions so that they maintain a minimal distance between QTLs similar to the one between real QTLs. We calculated the estimated variance explained by QTLs for each threshold, as the median variance explained by the 100 test set with the real QTLs, minus the median of the 10,000 sets with the random QTLs. It is important to note that random QTLs can be on top or in linkage with real QTLs, therefore our final estimation of variance explained by the real QTLs (after deduction of the variance explained by the random QTLs) might be an underestimation, especially for the low LOD detection thresholds where many regions of the genome are picked as QTLs and the chances that random QTLs are overlapping real QTLs is higher.

### Imputation

Of the 697 SNPs that were previously identified to be associated with height [3], only 145 were on our genotyping array. In order to impute the other 552 SNPs, we used the IMPUTE2 software [61,62], with two reference panels—the 1000 genomes (5008 phased haplotypes) [63] and a reference panel of Ashkenazi Jews (256 phased haplotypes) [35]. Despite the much smaller sample size of the Ashkenazi Jews panel, it yielded higher imputation accuracy as tested by the IMPUTE2 concordance tables (internal cross validation that IMPUTE2 conducts by imputing SNPs that are genotyped by the array). For SNPs that were confidently imputed by IMPUTE2 (maximum posterior probability ≥ 0.9) the concordance between the imputed and genotyped SNPs was on average 98% when using the Ashkenazi Jews reference panel, compared to 97% when using the 1000 Genomes reference panel.

Using the Ashkenazi Jews panel, we could impute 513 of the 552 missing SNPs, and the last 39 SNPs could be imputed by the 1000 Genomes reference panel.

## Supporting information

S1 Fig IBD reconstruction and correlations to height differences. **(a)** An example of IBD inference for two siblings, on a chromosomal segment. Dots represent similarity in genotypes between the two siblings—blue dots for all genotype calls, black dots for only high quality genotype calls. Opposite homozygous calls (e.g. sib 1 is AA and sib 2 is BB) are at the 0 alleles shared level. One homozygous and one heterozygous calls are at 1, and identical homozygous or identical heterozygous are at 2. Red lines represent the inferred IBD. (**b**) The inferred phased IBD for an entire family on a chromosomal segment. Siblings are ordered from tallest to shortest. Green and black segments represent the two grandparental haplotypes from the mother side. Blue and red represent the grandparental haplotypes from the father side (**c**) Example of a correlation between height and genotypes inherited from the mother at a specific genomic position (**d**) Same as (c) but for haplotypes inherited from the father.
(TIF)

S2 Fig Linkage analysis results for all families combined. (TIF)

S3 Fig Autosomal QTLs in 100 training sets, each set containing 2/3 of families in the cohort. (**a**) Median number of autosomal QTLs detected for each LOD detection threshold in the 100 training sets (blue), the median number of QTLs estimated to be false (red) and true (green) according to the permutation analysis. (**b**) Median false discovery rate calculated from the permutation analysis for each detection threshold (**c**) Median significance of the total number of detected QTLs for each threshold. Red line for P value = 0.05 level.
(TIF)

S4 Fig Height variance explained by QTLs. (**a**) Boxplots of variance explained by the detected QTLs in 100 test sets (blue), and variance explained by random QTLs (red). (**b**) Difference between the medians in (a). (**c**) Significance of a rank-sum test for a difference between the distributions in (a), shown as–Log10(P).
(TIF)

S5 Fig Height variance explained by QTLs when competing against whole genome GRM only (no GWAS GRM). (**a**) Boxplots of variance explained by the detected QTLs in 100 test sets (blue), and variance explained by random QTLs (red). (**b**) Difference between the medians in (a). (**c**) Significance of a rank-sum test for a difference between the distributions in (a), shown as–Log10(P).
(TIF)

S6 Fig Infinitesimal model simulations. Distribution of the correlation coefficients between chromosome length and variance explained per infinitesimal model simulation. The red line points to the Pearson r of the real data.
(TIF)

S7 Fig The effect of LOD scores over genomic distance through linkage. The average difference in LOD score is larger for more distant positions along a chromosome (blue line), up to a distance of ~33Mbps (green dotted line), where it reaches its median value (red dotted line), and then fluctuates around this value afterwards. Note that there are less data points to calculate the function as distance increases (e.g. fewer positions in the genome are 200Mbps apart than 100Mbps apart).
(TIF)

S1 Table Cohort information. (PDF)

S2 Table All detected QTLs. All genomic coordinates refer to genome build hg19.
(XLSX)
