# Supplementary figures and images for: Analysis of the genetic basis of height in large Jewish nuclear families
Source: PLoS Genet. 2019 Jul 8;15(7):e1008082. doi: 10.1371/journal.pgen.1008082 (PMC6638967; doi:10.1371/journal.pgen.1008082)

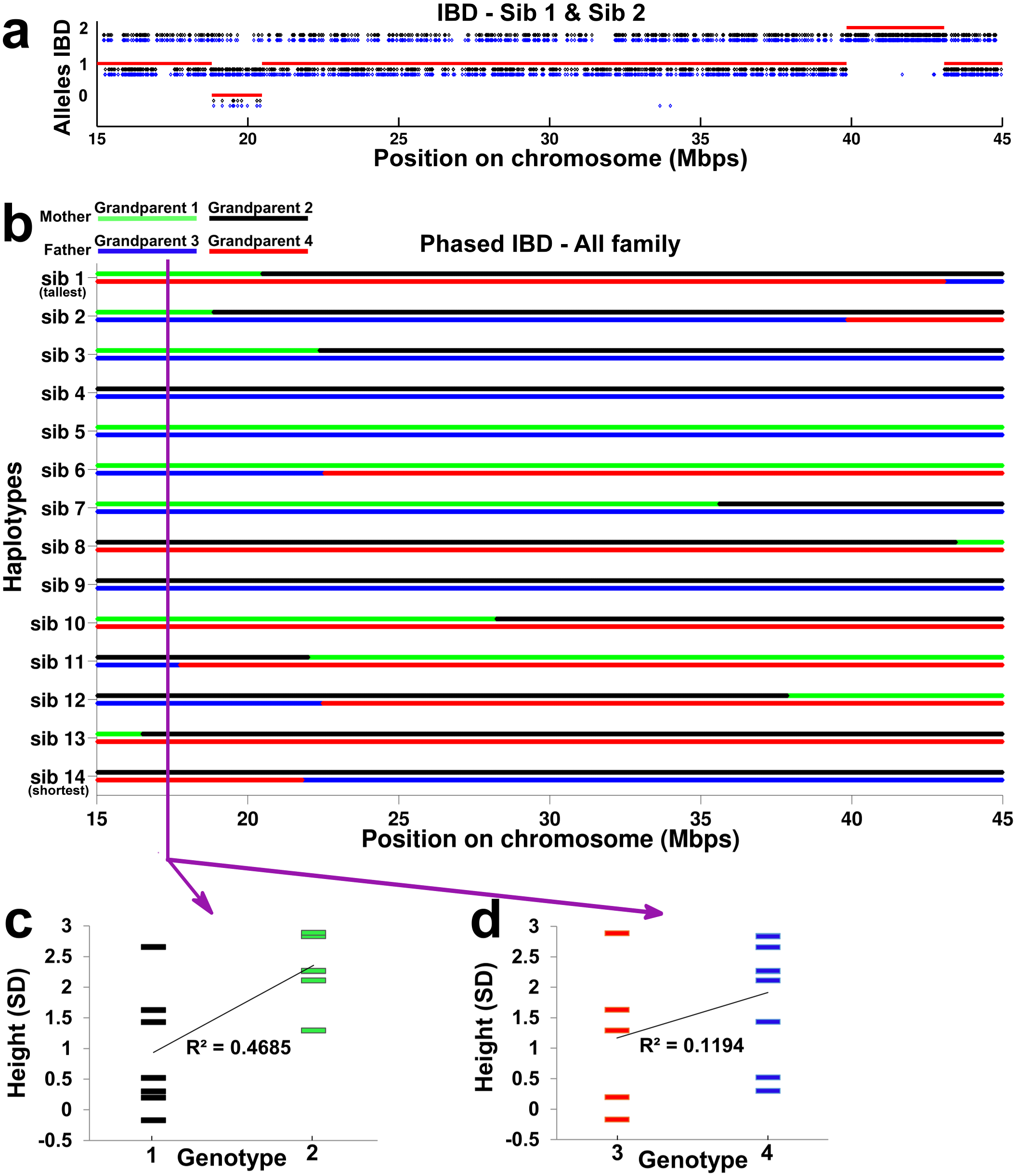

Supplement: S1 Fig — (a) An example of IBD inference for two siblings, on a chromosomal segment. Dots represent similarity in genotypes between the two siblings—blue dots for all genotype calls, black dots for only high quality genotype calls. Opposite homozygous calls (e.g. sib 1 is AA and sib 2 is BB) are at the 0 alleles shared level. One homozygous and one heterozygous calls are at 1, and identical homozygous or identical heterozygous are at 2. Red lines represent the inferred IBD. (b) The inferred phased IBD for an entire family on a chromosomal segment. Siblings are ordered from tallest to shortest. Green and black segments represent the two grandparental haplotypes from the mother side. Blue and red represent the grandparental haplotypes from the father side (c) Example of a correlation between height and genotypes inherited from the mother at a specific genomic position (d) Same as (c) but for haplotypes inherited from the father. (TIF) [file pgen.1008082.s001.TIF]

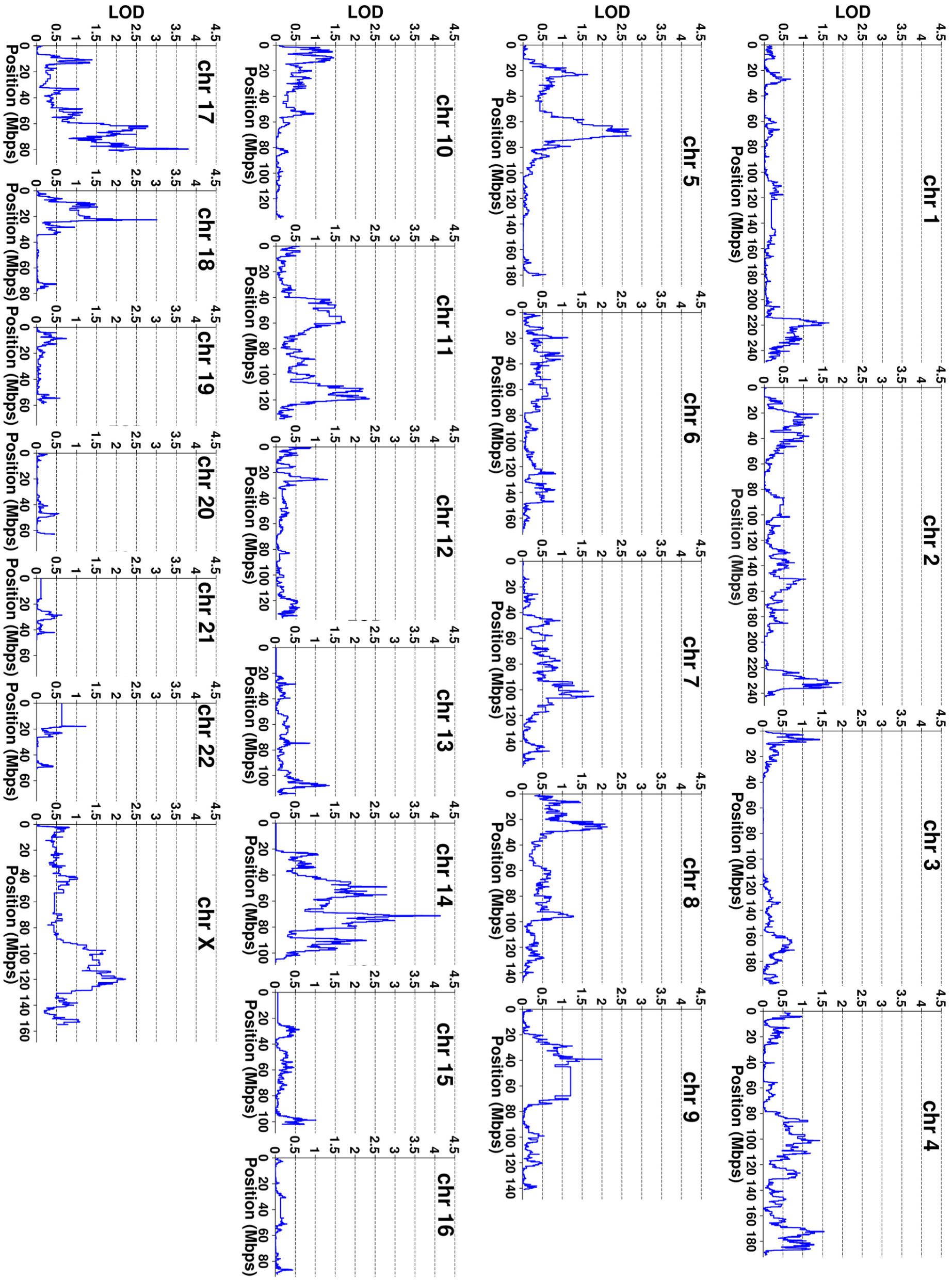

Supplement: S2 Fig — (TIF) [file pgen.1008082.s002.TIF]

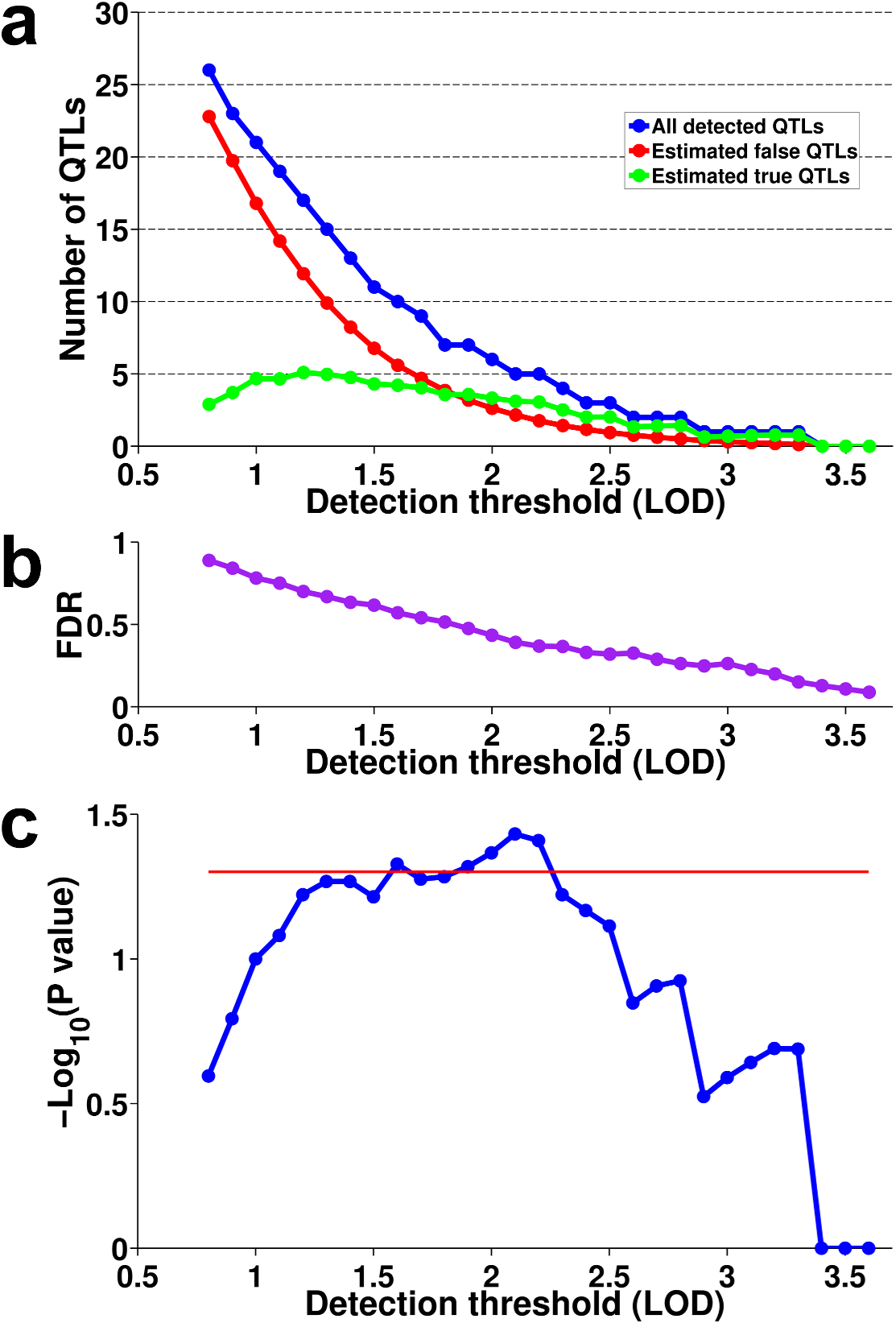

Supplement: S3 Fig — (a) Median number of autosomal QTLs detected for each LOD detection threshold in the 100 training sets (blue), the median number of QTLs estimated to be false (red) and true (green) according to the permutation analysis. (b) Median false discovery rate calculated from the permutation analysis for each detection threshold (c) Median significance of the total number of detected QTLs for each threshold. Red line for P value = 0.05 level. (TIF) [file pgen.1008082.s003.TIF]

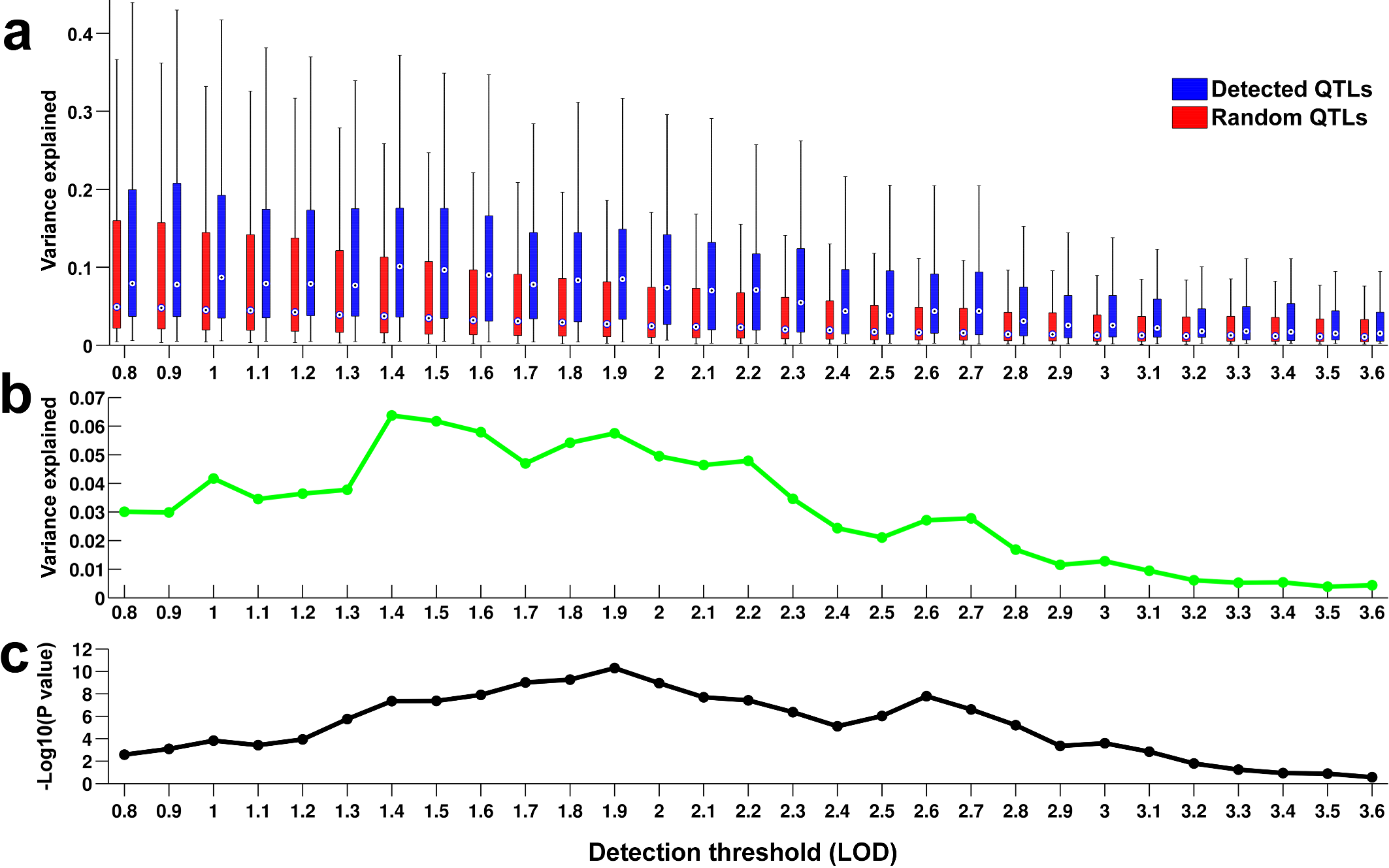

Supplement: S4 Fig — (a) Boxplots of variance explained by the detected QTLs in 100 test sets (blue), and variance explained by random QTLs (red). (b) Difference between the medians in (a). (c) Significance of a rank-sum test for a difference between the distributions in (a), shown as–Log10(P). (TIF) [file pgen.1008082.s004.TIF]

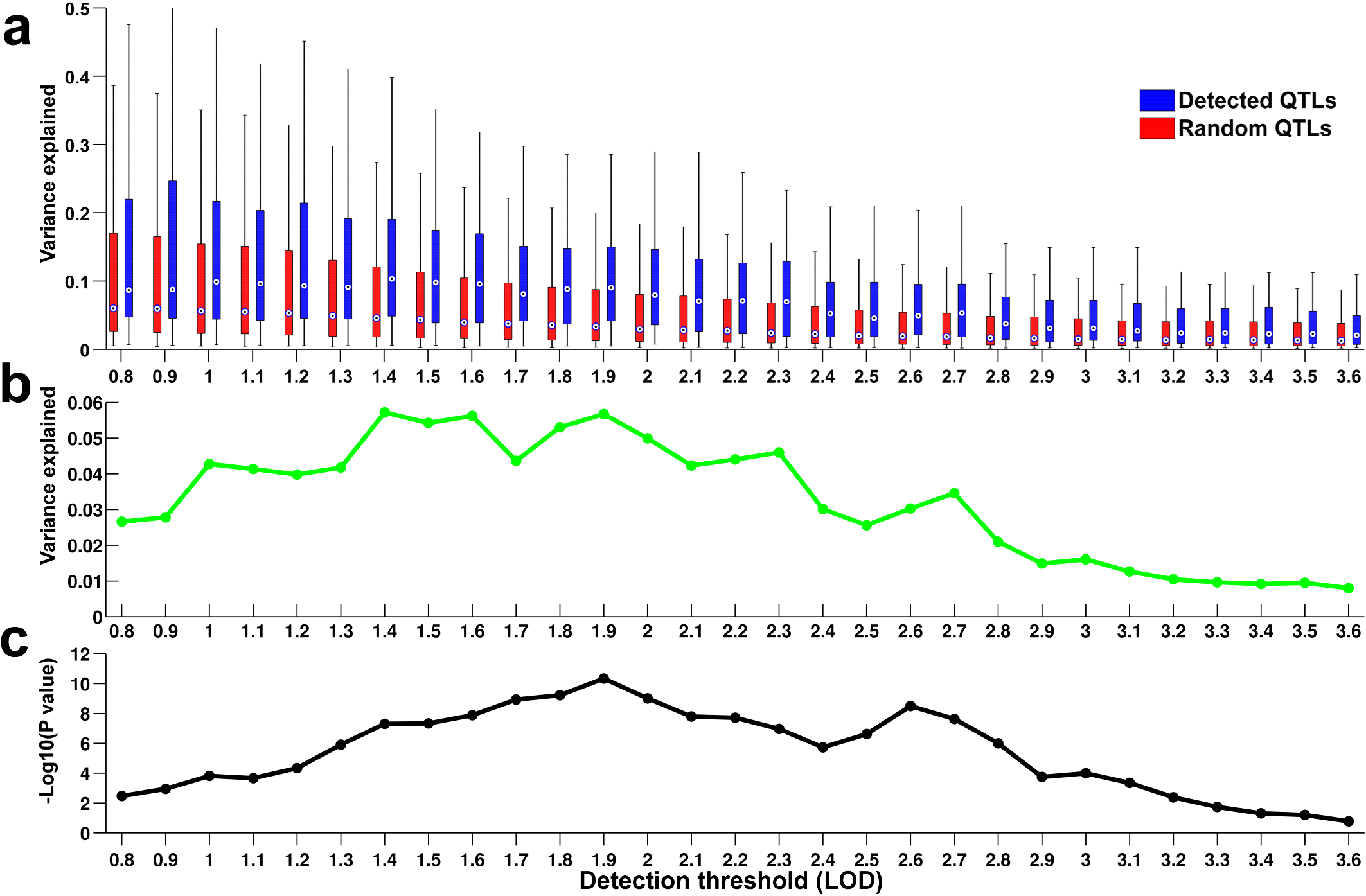

Supplement: S5 Fig — (a) Boxplots of variance explained by the detected QTLs in 100 test sets (blue), and variance explained by random QTLs (red). (b) Difference between the medians in (a). (c) Significance of a rank-sum test for a difference between the distributions in (a), shown as–Log10(P). (TIF) [file pgen.1008082.s005.TIF]

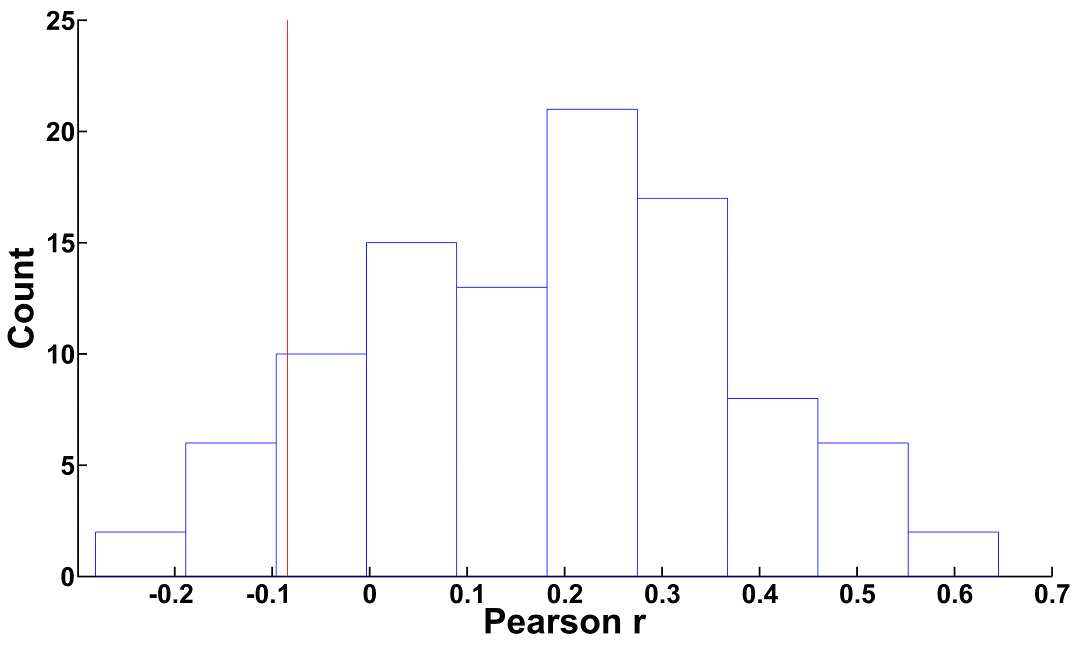

Supplement: S6 Fig — Distribution of the correlation coefficients between chromosome length and variance explained per infinitesimal model simulation. The red line points to the Pearson r of the real data. (TIF) [file pgen.1008082.s006.TIF]

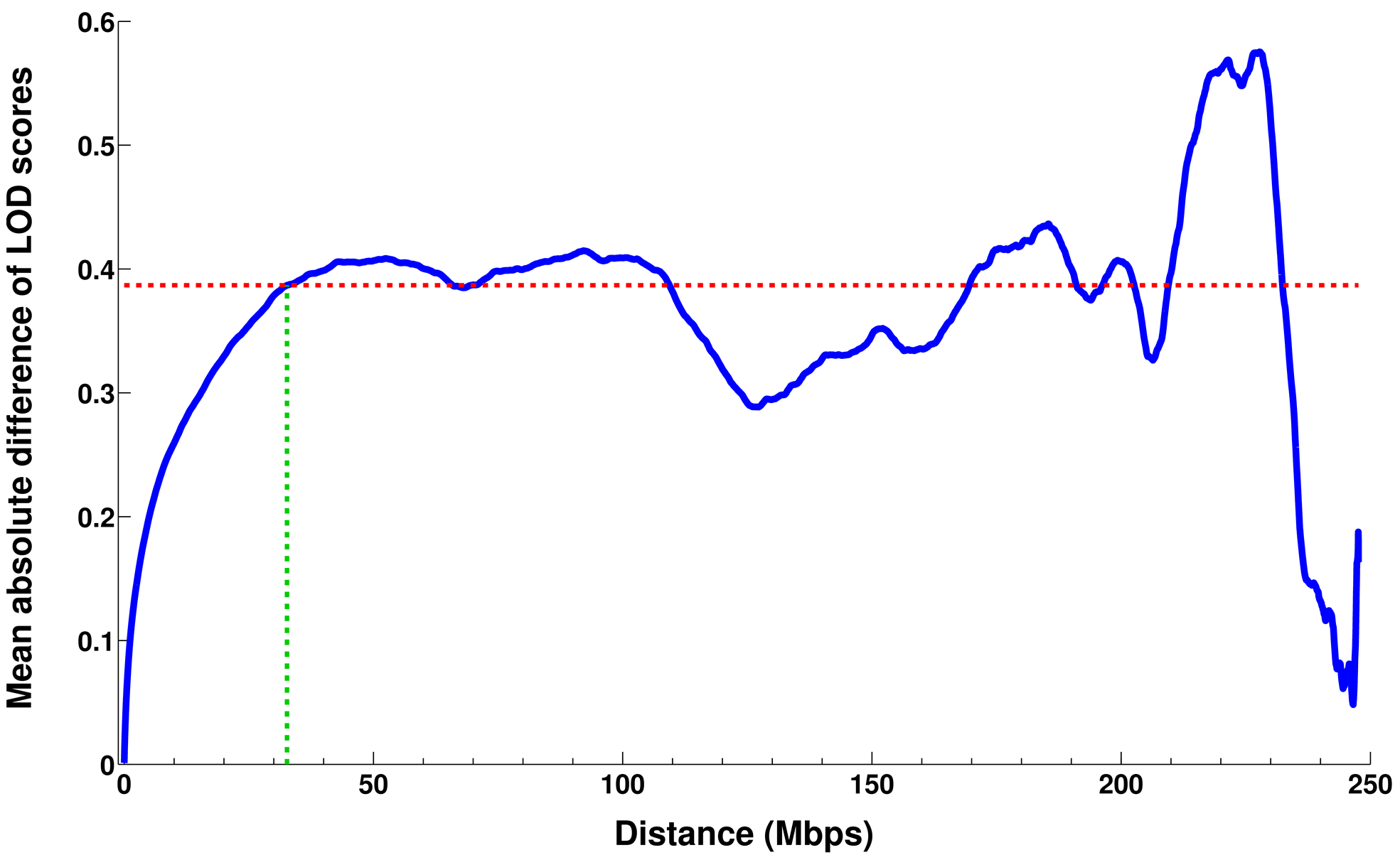

Supplement: S7 Fig — The average difference in LOD score is larger for more distant positions along a chromosome (blue line), up to a distance of ~33Mbps (green dotted line), where it reaches its median value (red dotted line), and then fluctuates around this value afterwards. Note that there are less data points to calculate the function as distance increases (e.g. fewer positions in the genome are 200Mbps apart than 100Mbps apart). (TIF) [file pgen.1008082.s007.TIF]
