# Supplementary material for: Analysis of the genetic basis of height in large Jewish nuclear families
Source: PLoS Genet. 2019 Jul 8;15(7):e1008082. doi: 10.1371/journal.pgen.1008082 (PMC6638967; doi:10.1371/journal.pgen.1008082)
Supplement: S1 Table — (PDF) [file pgen.1008082.s008.pdf]

S1 Table. Cohort information.

	Number of participants	Measured Height Mean±SD (cm)	Age Mean±SD (years)	Ashkenazi Jewish	Sephardi Jewish	Mixed Ashkenazi -Sephardi Jewish
Males	210	172.7±5.7	34.4±12.2	161	27	22
Females	187	161.6 ±5.5	35.7±13.2	151	24	12
Total	397	167.4 ±7.9	35±12.7	312 (78.6%)	51 (12.8%)	34 (8.6%)
